# Combinatorial nanocarrier based drug delivery approach for amalgamation of anti-tumor agents in bresat cancer cells: an improved nanomedicine strategies

**DOI:** 10.1038/srep34053

**Published:** 2016-10-11

**Authors:** Chandran Murugan, Kathirvel Rayappan, Ramar Thangam, Ramasamy Bhanumathi, Krishnamurthy Shanthi, Raju Vivek, Ramasamy Thirumurugan, Atanu Bhattacharyya, Srinivasan Sivasubramanian, Palani Gunasekaran, Soundarapandian Kannan

**Affiliations:** 1 Proteomics and Molecular Cell Physiology Laboratory, Department of Zoology, Periyar University, Salem-636011, TamilNadu, INDIA; 2 King Institute of Preventive Medicine & Research, Guindy, Chennai 600 032, Tamil Nadu, INDIA; 3 Department of Zoology, Bharathiar University, Coimbatore, 641 046, Tamil Nadu, INDIA; 4 Department of Biomedical Engineering, Shanghai Jiao Tong University, Shanghai, 200 240, CHINA; 5 Department of Animal Science, Bharathidasan University, Tiruchirappalli, 620 024, Tamil Nadu, INDIA; 6 Nanotechnology Section, Department of Biomedical Engineering, Rajiv Gandhi Institute of Technology and Research Centre, Hebbal, Bangalore, 560 032, Karnataka, INDIA

## Abstract

Combination therapy of multiple drugs through a single system is exhibiting high therapeutic effects. We investigate nanocarrier mediated inhibitory effects of topotecan (TPT) and quercetin (QT) on triple negative breast cancer (TNBC) (MDA-MB-231) and multi drug resistant (MDR) type breast cancer cells (MCF-7) with respect to cellular uptake efficiency and therapeutic mechanisms as *in vitro* and *in vivo*. The synthesized mesoporous silica nanoparticle (MSN) pores used for loading TPT; the outer of the nanoparticles was decorated with poly (acrylic acid) (PAA)-Chitosan (CS) as anionic inner-cationic outer layer respectively and conjugated with QT. Subsequently, grafting of arginine-glycine-aspartic acid (cRGD) peptide on the surface of nanocarrier (CPMSN) thwarted the uptake by normal cells, but facilitated their uptake in cancer cells through integrin receptor mediated endocytosis and the dissociation of nanocarriers due to the ability to degrade of CS and PAA in acidic pH, which enhance the intracellular release of drugs. Subsequently, the released drugs induce remarkable molecular activation as well as structural changes in tumor cell endoplasmic reticulum, nucleus and mitochondria that can trigger cell death. The valuable CPMSNs may open up new avenues in developing targeted therapeutic strategies to treat cancer through serving as an effective drug delivery podium.

Cancer is one of the most dreadful causes of death globally due to its increased incidence and mortality in recent years^1^. The developments in nanotechnology or Nanomedicine in drug delivery have opened up new arenas for designing efficient nanocarrier system/multifunctional nanomaterials for cancer therapy including combination therapies using nanoparticles like polymer micelles^2^,^3^,^4^,^5^. Combination therapy refers to the co-administration of multiple drugs through a single system, and the strategy is useful to exhibit high therapeutic effects, to overcome the undesirable side effects to normal tissues/cells as well as drug resistance and to improve survival rates of cancer therapy^6^,^7^,^8^. TPT is a hydrophilic anticancer drug that acts as a DNA topoisomerase 1 (TOP1) inhibitor to form a ternary cleavable complex between topo-I and the DNA, thus leading to the DNA cleavage and cell death (apoptosis). Despite its potency, the use of TPT in the cancer treatment is limited due to its poor water solubility, toxicity, and side effects^9^. Combination chemotherapy through targeted drug delivery systems promotes sustained release of hydrophobic small molecules specifically at tumor sites with reduced toxicity^10^.

Quercetin (3, 5, 7, 3′, 4′-pentahydroxyflavone) is a phytochemical^11^ having a wide range of chemotherapeutic activities against several diseases such as cancer, inflammatory, viral infections etc., besides possessing antioxidant properties^12^. Several reports are available on the potential of quercetin to inhibit the development and proliferation of diverse cancer cells, such as ovarian cancer, breast cancer, lung cancer, colon cancer, etc. The stimuli-responsive drug delivery systems are desirable in clinical medicine due to their enhanced therapeutic effectiveness and minimal adverse effects of the drugs. The pH-responsive drug delivery systems are the most investigated models since the pH values, notably vary in different tissues and cellular compartments as well as tumor microenvironments. The extracellular environment of a tumor is slightly acidic (pH ~6.8) than blood and normal tissues (pH ~7.4) while intracellular organelles such as endosome and lysosome are more acidic (pH ~5.0–5.5). Thus the pH-susceptible delivery systems are preferred for controlling drug-delivery^2^,^13^. Mesoporous silica nanoparticles (MSNs) are gaining more importance as an ideal drug container than polymers, micelles and liposomes owing to a tunable aperture size, great aperture volume, superior biocompatibility, and simple functionalization against cancer cells^14^,^15^,^16^. The use of inorganic nanoparticles^17^, organic molecules^18^, and biomolecules^19^ as MSN gatekeepers demonstrated well-controlled discharge performance^16^. However, the simultaneous and cascade discharge of two or more drugs is still difficult to accomplish. There is a necessity to develop high performance multidrug release nanosystems offering the combination therapy by targeting and controlled release of drugs. Targeted drug delivery systems use drug carriers that are linked with cell-specific targeting ligands, and moieties such as peptides, folic acid, glycyrrhetinic acid or antibodies can serve as targeting ligands depending upon the cell^2^,^4^,^16^,^20^,^21^,^22^. Arginine-glycine-aspartic acid (RGD) has been reported to recognize specific the ανβ3 and ανβ5 integrin receptors, which are over expressed in emerging endothelial cells during angiogenesis in diverse tumors^23^. The drug delivery vehicles grafted with cRGD peptides bind to over expressed αvβ3 integrins by tumor cells, resulting in receptor-mediated endocytosis and drug release by acidic endocytic organelles^24^. Nanomaterials are designed to possess features such as structural, biocompatibility, stability, and functionality if they are tailored with polymers. Polymer-based drug delivery materials facilitate high payloads, high shelf life of drugs, drug targeting ability of carriers and their solubility, and controlled release of the therapeutics into the blood stream or the targeted tumor tissues^25^.

Hence, in the present study, the PAA-CS polymers capped pH-responsive MSNs (CPMSNs) are designed with the properties of both targeted and combinatorial drug delivery against triple negative breast cancer cells (TNBCs). The developed MSNs are entrapped with a combination of drugs, initially the loading TPT in the pores by physical adsorption followed by surface coating of MSNs with polymers to block the pore as well as encapsulation of QT lead to avoid the release of cargoes due to reduction in external pH conditions. The MSNs functionalized with polymers (PAA-CS) that serve as gatekeeper as well as drug carrier and the conjugation of cRGD to the external surface of the polymer capped MSNs, assists targeted drug delivery through recognizing over expressed integrin receptor αvβ3 on tumor cell surface and improves shelf life of the synthesized nanosystem *in vivo*. The synthesized CPMSNs are stable at physiological pH (~7.4) but dissociate and release drugs in acidic pH (~5.5) of endocytic organelles of TNBCs upon receptor mediated endocytosis of the nanosytems. Studies are also performed to investigate the pH response drug release kinetics, stability, targeting and cellular uptake efficiency, bioavailability, synchronized delivery of co-encapsulated drugs, toxicity and anticancer capability of multifunctional CPMSNs using MDA-MB-231 and MCF-7 breast cancer cells along with HBL-100 cells (Control).

## Results and Discussion

### Synthesis and Characterization of CPMSN

**Figure 1:**
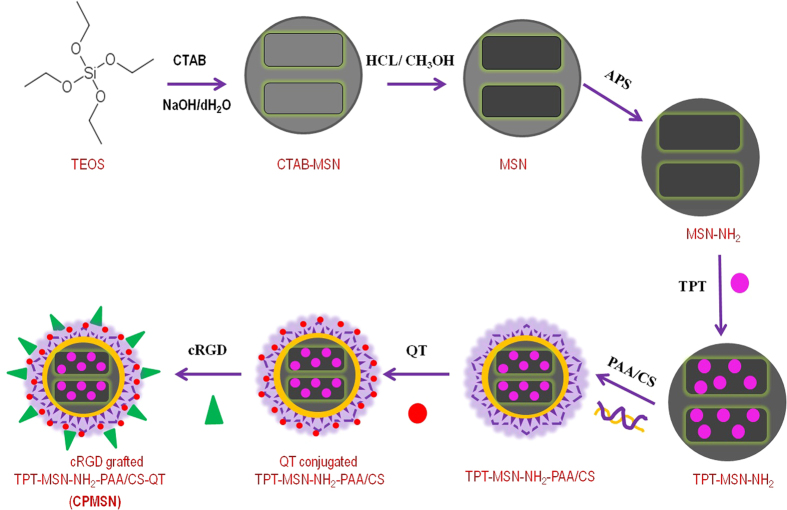
Schematic representation of process describing the preparation of cRGD peptide grafted CPMSNs nanocarrier. The surface of MSN nanocore is coated with pH sensitive polymers and the prepared CPMSNs act as potential tumor targeted dual drug delivery nanocarriers.

MSNs potentially mediate drug delivery against cancer cells due to their well defined structure and biocompatibility^26^,^27^,^28^. In this study, the MSNs were synthesized using an aqueous solution consisting tetraethyl orthosilicate (TEOS) and N-cetyltrimethylammonium bromide (CTAB) and functionalized with 3-Aminopropyltriethoxysilane (APS). The cationic topotecan (TPT) was loaded into the pores of MSNs, which rich in anionic silanol, via electrostatic interaction. TPT loaded MSN channels were then capped with the PAA-CS layers to facilitate pH dependent activation of the nanomaterials and to entrap quercetin (QT) as a second drug. To enable cancer cell targeting of the drug loaded nanoparticles, cRGD peptide was covalently conjugated to the outer layer of PAA-CS as results that the formed CPMSNs nanocarrier. It is well known that the cRGD peptide specifically recognizes cancer cells through cRGD-integrin interactions^29^. Figure 1 describes the synthesis of CPMSN and it was used to target the cancer cells via the interaction with integrins, which were found to be overexpressed on the surface of various cancer cells^30^,^31^.

**Figure 2:**
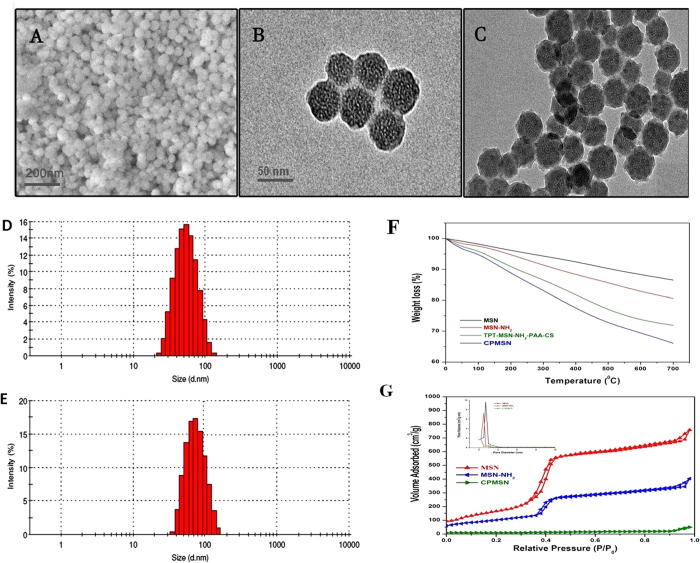
The analysis of morphological features of synthesized nanomaterials by electron microscopy, DLS and TGA methods. (**A**) Spherical shape of the MSNs is shown by SEM analysis; TEM studies reveal (**B**) the mesoporous amorphous nature of MSNs and (**C**) CPMSNs microspheres; DLS studies on size distribution of (**D**) MSNs and (**E**) CPMSNs; (**F**) TG analyses show decreasing curve trends for MSNs, MSNs-NH_2_, TPT-MSN-NH_2_-PAA-CS and CPMSNs and (**G**) nitrogen adsorption desorption isotherms authenticate the porous nature of synthesized MSNs, MSN-NH_2_, and CPMSNs.

Scanning electron microcopy (SEM) analysis was performed to study the morphology of MSNs. Figure 2A reveals the monodisperse and the roughly spherical shape of MSNs. Transmission electron microscopy (TEM) image (Fig. 2B) shows that the MSNs have a uniform and well defined mesopores and the size of MSNs is found to be approximately between 45–55 nm. TEM image of CPMSNs (Fig. 2C) shows a thin lucid outer layer implying the presence of organic moieties that completely covers the MSNs core. The sizes of CPMSNs are in the range of 65–75 nm which is slightly larger than MSNs. Besides, the nonporous nature of the particle surface indicates that the nano-cavities of the drug carriers are filled with drug moieties. The average hydrodynamic diameter and size distribution of MSNs and CPMSNs dissolved in deionized water were measured by dynamic light scattering (DLS) at 37 °C. As shown in Fig. 2D, the diameter of MSNs was 53.2 nm with a polydispersity index (PDI) of 0.01 whereas CPMSNs (Fig. 2E) had a larger diameter of 72.9 nm with a PDI of 0.03. These results suggested that the nanocarriers having a diameter less than 200 nm could effectively target tumors *in vivo* through the enhanced permeability and retention (EPR) effect^32^.

Thermogravimetric analysis (TGA) was used to determine the residual components in CPMSN based on the examination of weight loss of individual components in the nanomaterials after each modification. It is observed from the TGA curves that when the temperature is elevated to 700 °C, the weight loss of blank MSNs, MSN-NH_2_, TPT-MSN-NH_2_-PAA-CS and CPMSN are found to be ~13.5%, 19.4%, 28.1%, and 33.9% respectively (Fig. 2F). The nitrogen adsorption/desorption isotherm and pore volume of MSN; MSN-NH_2_ and CPMSN are presented in Fig. 2G and the results indicated the porous nature of the synthesized nanomaterials. SBET (specific surface area Brunauer–Emmett–Teller) and the total pore volume (Vt) of MSN were 843 m^2^g^−1^ and 0.892 cm^3^/g, respectively. After functionalization of MSN with APS, SBET and Vt of MSN-NH_2_ were 675 m^2^g^−1^ and 0.843 cm^3^/g, respectively. The decrease in surface area and pore volume of the amine functionalized nanoparticles (MSN-NH_2_) compared with the MSNs was due to the presence of organic groups occupying the pore spaces in the MSNs. Furthermore, the values of SBET and Vt were drastically reduced to 118.0 m^2^g^−1^ and 0.186 cm^3^/g, respectively in CPMSNs indicating the loading of drug molecules into the mesoporous channels and subsequent functionalization of the MSNs with each component. Besides, the analysis of pore size distribution of MSN, MSN-NH_2_ and CPMSN using the Barrett-Joyner-Halenda (BJH) method clearly shows that the MSN exhibits an intensive pore diameter peak at 2.7 nm, which is reduced to 2.5 nm after functionalization with APS indicating the effect of APS on pore blocking, however the pore volume of MSN-NH_2_ was still large enough for drug loading (Fig. 2G). These results demonstrated that the drug molecule TPT was successfully loaded into the pores of MSN-NH_2_ that were subsequently functionalized with polymer PAA-CS, QT and cRGD peptides to obtain multifunctional tumor targeting CPMSNs.

The surface functionalization of CPMSN was evaluated by fourier transform infrared (FT-IR). The FT-IR spectra of MSN-CTAB, MSN, MSN-NH_2_, TPT-MSN-NH_2,_ TPT-MSN-NH_2_-PAA-CS, TPT-MSN-NH2-PAA-CS-QT and CPMSN are shown in Figure S1. The spectra of MSN-CTAB showed both C-H stretches vibrations at 2922 cm^−1^ and 2852 cm^−1^ and C-H deformation vibrations at 1474 cm^−1^ due to the presence of CTAB. However, the removal of CTAB from MSN-CTAB resulted in disappearance of C-H absorbance peaks attributed to CTAB and appearance of strong absorption signals at 1080 cm^−1^ and 954 cm^−1^, which were assigned to asymmetric stretching of Si-O-Si bridges and skeletal vibration of the C-O stretching, respectively. MSN-NH_2_ displayed additional peak at 1582 cm^−1^, which was assigned to the stretching vibration of -NH_2_ bending. The appearance of C-H stretching vibrations at 2929 cm^−1^ confirmed the successful functionalization of MSNs with amino groups. The loading TPT was confirmed by the absorption peaks at 1745 cm^−1^ assigned to ester carbonyl stretching vibration. After polymer (PAA-CS) coating of nanomaterials, several new adsorption peaks related to PAA appeared at 1556 cm^−1^, 1655 cm^−1^ and 1718 cm^−1^, which could be assigned to the N-H bending vibration, C=O stretching vibration in the amide group and C=O stretching vibration in the carboxyl group, respectively. Absorption peaks of chitosan at 1666 cm^−1^ and 1586 cm^−1^ were attributed to the amide bonds, indicating the successful coating of PAA-CS on TPT-MSN-NH_2_. The conjugation of QT to the matrix of TPT-MSN-NH_2_-PAA-CS was confirmed by the appearance of peak at 1451 cm^−1^ and 1200 cm^−1^. After cRGD grafting on PAA-CS membranes of TPT-MSN- NH_2_-PAA-CS-QT, the characteristic peak at 1586 cm^−1^ disappears indicating an interaction in the primary N-H bending region. This result suggested the formation of a covalent bond between cRGD and the primary amino group of PAA-CS. In addition, the characteristic IR absorption peak at 1385 cm^−1^ (amide III and C–N stretch vibration) of cRGD peptides was found in the spectra of CPMSN indicating the successful binding of peptide molecules to the TPT-MSN-NH_2_-PAA-CS-QT. These results validated the grafting of cRGD on the PAA-CS membranes and successful synthesis of CPMSNs.

The prepared MSN, MSN-NH_2_ and CPMSN were also investigated by Zeta (ζ) potential analysis (Table 1). The zeta potential of MSN was −20.4 mV and after surface modification of MSNs with amino groups (MSN-NH_2_), it was +16.4 mV. The zeta potential of CPMSN was +42.8 mV and this change was mainly due to the presence of amino groups in the backbone of CS and the cationic TPT loaded in the pores of MSNs.

### Drug Loading Profile of CPMSNs

The loading of TPT and QT on CPMSNs is based on the electrostatic and hydrophobic interactions, respectively. Cationic TPT is lodged into the pores of MSNs that contain negatively charged SiO^−^ groups at physiological pH. On the contrary, the hydrophobic drug QT is poorly soluble in aqueous solution. The polymer PAA-CS has both hydrophilic and hydrophobic segments, and QT can be trapped into its core region. The drug loading content of CPMSN (mass of drug in the CPMSNs/mass of CPMSNs loaded with drug × 100%) and encapsulation efficiency (mass of drug achieved in the NCs/mass of the feeding drug × 100) 0.010/0.014 × 100 = 71.4% of TPT and QT 0.012/0.018  × 100 = 66.6%) of TPT and QT in CPMSN were calculated as 1.8, 1.2 wt%, respectively.

### *In vitro* Drug Release

**Figure 3:**
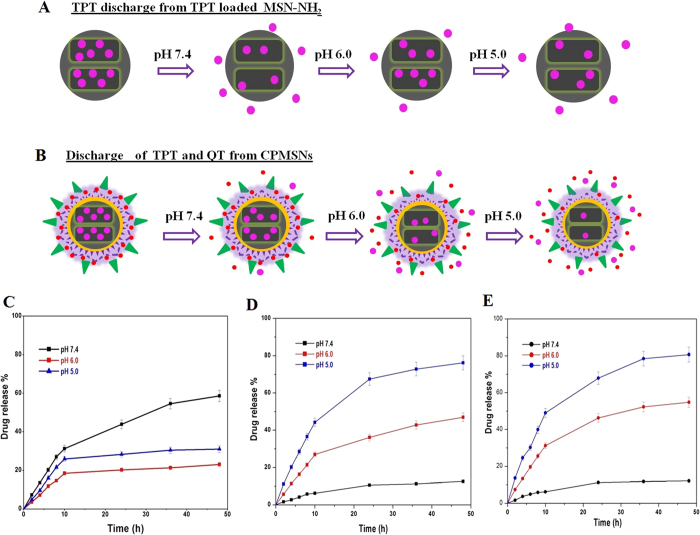
Schematic representation of pH-dependent drug release profile of TPT loaded MSN-NH_2_ and CPMSNs were examined under the physiological pH (7.4) and tumor environmental pH (6.0) and endo/lysosomal pH (5.0) at 37 °C (**A,B**). Drug release curves (**C**) for TPT from MSNs-NH_2_, (**D**) TPT released from CPMSNs and (**b**) QT released from CPMSNs. Error bars are based on mean ± SD of triplicate measurements.

Controlled and sustained *in vitro* drug release profile from MSN-NH_2_ and CPMSNs were obtained by dialysis experiment on drug release conducted at different pH *viz.* pH 7.4, pH 6.0 and pH 5.0 correspond to the circulation system, tumor environment and mature endosomes of tumor cells, respectively at ambient temperature (37 °C) (Fig. 3). Figure 3A shows the pH-responsive release of TPT from TPT-MSN-NH_2_ without the coating of PAA-CS polymers. The release rates of TPT in solutions of pH 7.4, pH 6.0 and pH 5.0 in 48 h were 58.63%, 23% and 29.99%, respectively (Fig. 3C). At pH 7.4, the carboxylic group of TPT exhibited an electrostatic repulsion with SiO^−^ in the pores of MSN resulting in the release of high quantity of drug. However, at pH 6.0, due to weak electrostatic interaction of SiO^−^-TPT resulted in discharge of anticancer drug TPT. Further reduction in pH 5.0 contributed to electronegativity of SiO^−^, which was closer to the silica isoelectric point; however, the electropositivity of TPT was slightly improved which causing weak electrostatic interaction of SiO^−^TPT^28^ to release more amount of TPT from TPT-MSN-NH_2_ than that of the solution having pH 6.0. Hence, the pH-sensitivity of MSN materials was not adequate and desirable. At pH 7.4, the discharge of TPT from CPMSN was very slow (8.7%) while the release of QT was slightly higher (12.03%) in 48 h (Fig. 3D,E) and this indicated that PAA-CS coated MSNs were stable under the physiological condition. Nevertheless, a burst release of TPT and QT from CPMSN was observed; about 46.98% with 54.77% of TPT and QT were released at pH 6.0 and 76.24% and 80.72% of TPT with QT were released at pH 5.0 during the 48 h at ambient 37 °C. The controlled drug release of TPT and QT could be attributed to similar release mechanisms. The release of TPT and QT was controlled by the pH sensitive polymers PAA-CS layered densely on the surface of nanoparticles. PAA-CS polymers entrap QT in its matrix and also block the pores, thus preventing the release of QT from the matrix and TPT from the pores at pH 7.4, On the contrary, the hydrolysis of PAA-CS leads to simultaneous dissociation of the polymer on the surface of the nanocarrier at low pH (pH 6.0 and 5.0). Subsequently, it enhances the release of QT from polymer and TPT release from the pores of MNPs as well. This result suggested that the pH-responsive polymer PAA-CS was capable to release drugs from CPMSNs in controlled and sustainable manner. The premature release of TPT and QT from CPMSNs at pH 7.4 was found to be low/limited, and this would reduce the undesired effects in healthy tissues while these nanocarrier are in circulation. After the CPMSN reach the tumor site, the targeting property of cRGD peptide could be interacting with integrin receptor, which enabled the easy uptake of CPMSN nanocarrier by cancer cells and subsequent the acitic endosomal environment stimulates the hydrolysis of PAA-CS polymers induced intracellular drug release. The pH-sensitive drug release profile of this study is in corroboration with the previous report on the TPT release from MSNs^28^. In the previous study, the different kinds of water soluble polymers can be tailored to exhibit the external stimuli such as temperature and pH, which used to sustained release of therapeutics at the cellular levels, this can also afford as injectable materials in drug delivery and tissue engineering. Overall, the combination drug loaded nanocarrier systems might be useful for and considered to specifically bind to α_v_β_3_ integrin expressed on endothelial cells in the angiogenic blood vessels, which inhibits the tumor growth. Hence, these pH-sensitive polymer nanocarrier have appeared to be a promising alternative system for tumor-targeted delivery of TPT-QT as in combination on the desired site intracellularly. These kinds of novel developments may revise the classical outlook that nanocarriers which are passive delivery vehicles, in favor of respondents, sensing vehicles that use environmental cues to achieve maximal drug potency against cancer^33^,^34^,^35^,^36^,^37^,^38^.

### Cytotoxicity Studies

**Figure 4:**
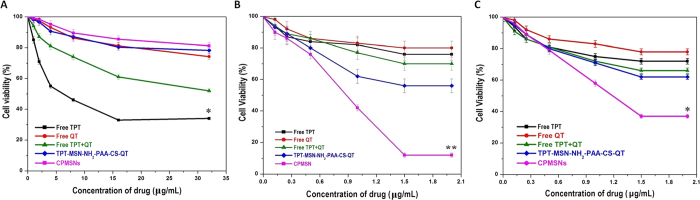
Evaluation of cell viability by MTT assay. (**A**) HBL-100, (**B**) MDA-MB-231 cells and (**C**) MCF-7 cells treated with free TPT, free QT, free TPT+QT, TPT-MSN-NH_2_-PAA-CS-QT, and CPMSNs for 48h at various concentrations. The viability of MDA-MB-231 and MCF-7 cells treated with CPMSN carrying both the drugs was found to be significantly decreased when compared to cells treated with free drugs, combined TPT and QT treatment and TPT-MSN-NH_2_-PAA-CS-QT. The data represent mean ± SD. *p ≤ 0.05 was considered significant.

The cytotoxic effects of free TPT, free QT, free TPT+QT, TPT-MSN-NH_2_-PAA-CS and CPMSNs were evaluated on human breast cancer cell lines, such as MDA-MB-231 and MCF-7 and normal breast cell lines (HBL-100) by MTT assay. The treatment of HBL-100 by free TPT, free QT and free TPT+QT, TPT-MSN-NH_2_-PAA-CS-QT at different drug concentrations in the range of 1–32 μg/mL showed no appreciable deduction in cell viability in 48 h incubation. It was shown that the increased concentration of nanoformulations exhibited less toxic effect than free drugs, indicating the biocompatible nature of CPMSNs (Fig. 4A). However, the treatment of cancer cells MDA-MB-231 and MCF-7 by free TPT, free QT, free TPT+QT, TPT-MSN-NH_2_-PAA-CS-QT, and CPMSNs at different drug concentrations (0.125–2.0 μg/mL) for 48 h resulted in increased cytotoxicity in a dose dependant manner (Fig. 4B,C). The results show that free drugs (TPT, QT) significantly inhibit the proliferation of MCF-7 cell when compared to MDA-MB-231. When the drugs are combined (TPT+QT), significant reduction in the viability of MDA-MB-231 and MCF-7 cells was observed while compared with free drug (P < 0.001). These findings on dual drug therapy against cancer cells were agreeing with the previous report^39^. It was reported that the concentration of the mesoporous nanoparticles as drug platforms to kill cancer cells was lower than 10 μg/mL^−1^ 40. Furthermore, treatment with TPT-MSN-NH_2_-PAA-CS-QT showed a remarkable inhibition in the proliferation of MDA-MB-231 and MCF-7 cell lines. The results denote that loading of drugs into the nanocarrier enhanced its cytotoxic effect when compared to free drugs. On the other hand, treatment with CPMSNs showed that the cytotoxic effect was higher in MDA-MB-231 (88.01%) cells than in MCF-7 (63%) cells, suggesting the overexpression of integrin receptor in MDA-MB-231 cells when compared to MCF-7 cells. This difference in cytotoxicity was mainly due to the targeting ability of CPMSNs to the cancer cells expressing integrin receptors. The cRGD peptide of CPMSNs actively binds to the overexpressed ανβ_3_ integrins on MDA-MB-231 breast cancer cells, leading to the rapid internalization of nanocarriers through receptor mediated endocytosis^41^,^42^. Further, the results shows that the CPMSNs were more readily internalized through an endocytosis mechanism than other MSNs due to possessing the positively charged CS and cRGD peptide. Upon internalization, CPMSNs release TPT and QT in the cytoplasm and enhance the therapeutic effect through exhibiting synergistic anti-proliferative activities on MCF-7 and MDA-MB-231 cell lines at an IC_50_ concentration of 1.18 μg/mL and 0.881 μg/mL, respectively.

### Internalization and distribution of dual drug loaded CPMSNs *in vitro*

**Figure 5:**
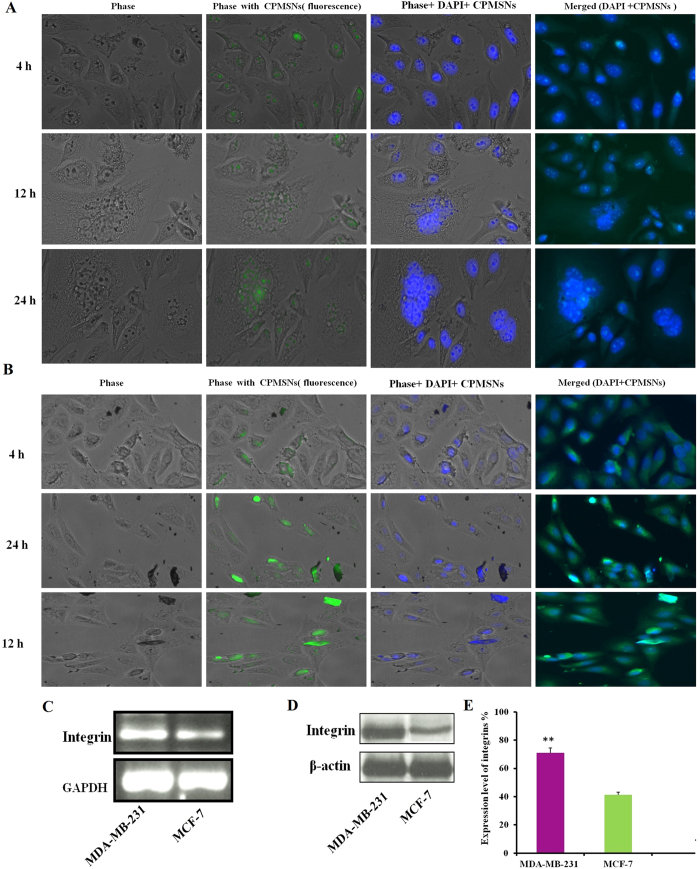
Cellular uptake efficacy and intracellular localization of CPMSNs were observed by confocal laser scanning microscopy. Figure (**A**) MDA-MB-231 and (**B**) MCF-7 cells treated with CPMSNs for various duration showed receptor mediated cell internalization of developed nanocarriers. The expression of integrin receptors on the cells was also demonstrated by (**C**) RT-PCR and (**D**) Western blot analysis. (**E**) The expression levels were also measured using densitometry profiles. Scale Bar 50 μm. *p ≤ 0.05.

The cellular uptake efficiency of CPMSNs was investigated in MDA-MB-231 and MCF-7 cells using laser confocal scanning microscopy. Various tumor cells, including breast cancer cells overexpress integrin receptors, which specifically recognize RGD peptides^29^,^31^. Significant cellular uptake of CPMSNs in MCF-7 and MDA-MB-231 cells was observed as evidenced by the green and blue fluorescence due to FITC staining of the cytoplasm and DAPI staining of the nucleus respectively (Fig. 5A,B). Moreover, the colocalization of green and blue fluorescence was clearly observed for in cells that were incubated with CPMSNs (IC_50_ concentration) for 4 h, 12 h, and 24 h, indicating that the CPMSNs were mainly localized within the cytoplasm after internalization. On the other hand, the cellular uptake was significantly enhanced in MDA-MB231 cells when compared to the MCF-7 cells due to overexpression of integrin receptors by MDA-MB-231 cells that recognized more CPMSNs. In addition, it was observed that the accumulation of CPMSNs by cells was high when cells were incubated for long duration. The expression of integrin receptors was demonstrated by RT-PCR, Western blot and densitometry studies, and it was observed that the expression was more in MDA-MB-231 cells than MCF-7 cells (Fig. 5C–E). Our findings were consistent with the results of previous studies reporting on the increased levels of integrin expression in triple negative breast cancer^43^,^44^. The specific characteristics of the tumor microenvironment is a major focus and possible to design drug delivery systems that specifically target anti-cancer drugs to tumors. Most of the conventional chemotherapeutic agents have poor pharmacokinetics profiles and are distributed non-specifically in the body, leading to systemic toxicity associated with serious side effects. Hence, the progress of drug delivery systems using a combinatorial approach which is conjugated with cRGD peptide can able to target the tumor site. It is becoming a real challenge that is currently addressed in the present work. These kinds of nanomedicine can reach the tumor passively through the leaky vasculature surrounding the tumors by the Enhanced Permeability and Retention effect, whereas ligands grafted at the surface of nanocarriers allow active targeting by binding to the overexpressed receptors by cancer cells for effective drug delivery approach. Here, we highlighted the design of smart nanosystems which release drugs in response to an intracellular biological signal such as acidic pH in endo/lysosomal compartments of triple negative breast cancer cells and to induce redox potential in the cytoplasm and the nucleus damages of apoptosis^45^,^46^,^47^,^48^.

### Analysis of Δψm loss and nuclear morphology by fluorescence microscopy

**Figure 6:**
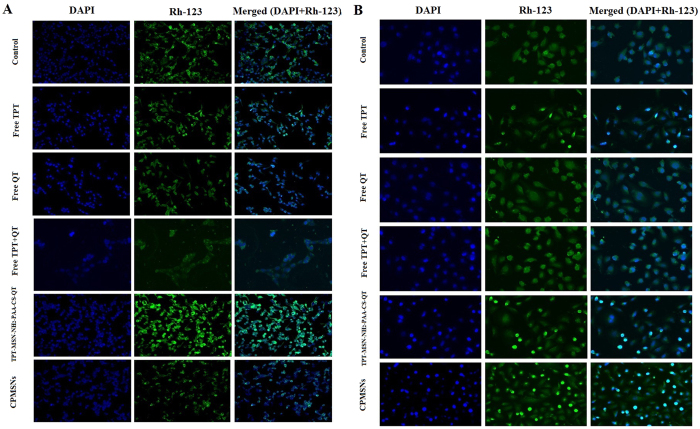
Fluorescence images (400x magnifications) of DAPI and Rh-123 stained (**A**) MDA-MB-231 and (**B**) MCF-7 cells treated with drugs and different nanoformulations for 48 h. The treated both cancer cells showed abnormalities in nucleus as well as mitochondrial membranes indicating the induction of apoptosis and loss of mitochondrial membrane potential (Δψm).

The effects of CPMSNs on nuclear condensation, Δψm loss, fragmentation of nucleic acid materials, and the morphology of MDA-MB-231 and MCF-7 cells were investigated by fluorescence microscopy (Fig. 6A,B). The fluorescent dye DAPI is known to form adducts with double-stranded DNA and the staining of cells with DAPI is used to determine the nuclear condensation in apoptotic cells. The results reveal that the drug treated cells have the abnormal features such as condensed morphology of nucleus as well as nuclei chromatins, gathering of nuclei chromatin at the periphery of the nuclear membrane and a completely fragmented morphology of nuclear bodies and these features indicate the possibility of induction of apoptosis. It was observed that the growth of cells was inhibited when they were treated with free TPT, free QT, free TPT+ QT, TPT-MSN-NH2-PAA-CS-QT, and CPMSNs at their final concentrations (2 μg/mL), and the CPMSNs possess higher antiproliferative efficacy against cancer cells than free drugs. These results suggest that CPMSNs could efficiently inhibit cancer cell proliferation as reported in a previous study^49^.

Rh-123 is used as tracer dye due to its ability to form fluorescent complexes with active mitochondrial membranes, was employed to determine the loss of mitochondrial membrane potential (Δψm) in breast cancer cells (Fig. 6A,B; middle column). The rate of Rh-123 fluorescence decay is directly proportional to the mitochondrial membrane potential. Fluorescence images of cancer cells show that there was a reduction in the mean fluorescence intensity for cells treated with free TPT, free QT, free TPT+QT, TPT-MSN-NH2-PAA-CS-QT, and CPMSNs confirming the loss of Δψm owing to mitochondrial membrane depolarization, which was considered to be an initial and irreversible step of apoptosis. The results further demonstrated that the loss of Δψm was higher in MDA-MB-231 cells than MCF-7 cells, indicating the effective internalization of CPMSNs and subsequent induction of apoptosis. Mitochondria play an important role in induction of intrinsic apoptotic pathway by releasing Cytochrome c for activation of the caspase cascade^50^. CPMSNs of the study can interrupt the functions of mitochondria through membrane depolarization leading to release of Cytochrome c to induce apoptosis in cancer cells. The merged DAPI and Rh-123 staining cells showed significant morphological changes in cells (green+blue color) due to the diminished size of apoptotic nuclei, assembly of condensed chromatin at the periphery of the nuclear membrane and loss of Δψm (Fig. 6A,B; right column). Hence, CPMSNs can enter inside the cells and distribute drugs to injure the nucleus and mitochondria more efficiently than free drug forms^51^,^52^.

### ROS generation in CPMSNs treated breast cancer cells

**Figure 7:**
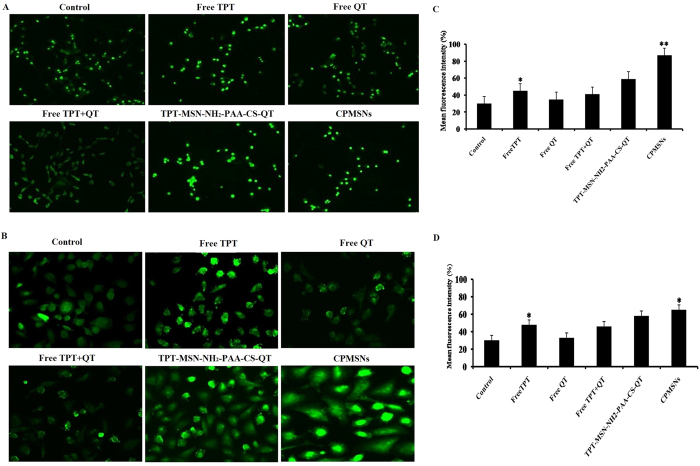
Fluorescence images (400x magnifications) of DCFH-DA stained (**A**) MDA-MB-231 and (**B**) MCF-7 cells showed the effect of drugs and different nanoformulations on ROS generation due to loss of Δψm. CPMSNs treated cells stained with DCFH-DA showed increased fluorescence intensity of DCF, which is proportional to the amount of ROS produced by the (**C**) MDA-MB-231 and (**D**) MCF-7 cells. The Data represent mean ± SD *p  ≤ 0.05 was considered statistically significant.

ROS generation were examined (Fig. 7A,B) in both cancer cells after treating them with free TPT, free QT, free TPT+QT, TPT-MSN-NH2-PAA-CS-QT and CPMSNs (2.0 μg/mL). ROS scavenger DCFH-DA stained treated MDA-MB-231 and MCF-7 cells showed increased fluorescence intensity when compared to untreated cells, suggesting that the chemotherapeutic drugs distributed by CPMSNs were able to ROS generation as well as DNA damage leading to induce apoptosis^53^. However, the TPT+QT drug combination did not show a statistically significant impact on ROS generation and this could be due to the antioxidant properties of QT. Though there was an increase in fluorescence intensity of cells treated with TPT-MSN-NH2-PAA-CS-QT and CPMSNs, cells treated with CPMSNs exhibited higher intensity of fluorescence than TPT-MSN-NH2-PAA-CS-QT. This suggested that the CPMSNs were the most valuable materials in the study to effectively induce the production of ROS in a concentration-dependent manner to activate intrinsic apoptotic signalling pathways in breast cancer cells. ROS intensity was analyzed by fluorescence plate reader (Fig. 7C,D) in both breast cancer cells, after the treating with drugs/ nanoformulation the higher fluorescence intensity was observed in CPMSNs treated MDA-MB-231 cells while compared with MCF-7 cells. Hence, the increased levels of ROS play a central role in the regulation of cell apoptosis. Generation of ROS subsequently induces ER stress, DNA damage and genomic instability, loss of Δψm and cell death^53^,^54^,^55^.

### Cellular internalization of CPMSNs by TEM analysis

**Figure 8:**
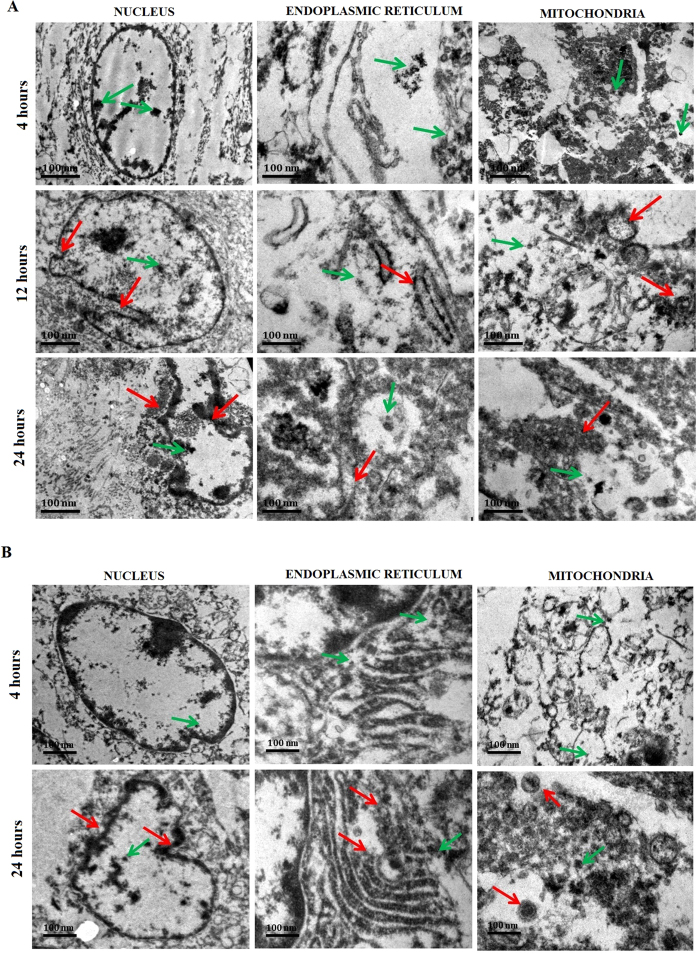
TEM images of cell organelles *viz*., nucleus, endoplasmic reticulum and mitochondria of (**A**) MDA-MB 231 and (**B**) MCF-7 cells treated with CPMSNs show the accumulation of nanocarriers (red arrows) as well as dislocations of cellular components or damaged cell organelles (green arrows) after different time intervals.

TEM analysis was performed to study the intracellular distribution of nanocarrier and the ultrastructural features of cancer cells treated with CPMSNs (Fig. 8A,B). The uptake of CPMSNs by MCF-7 and MDA-MB-231 cells through endocytosis, which leads to the sustained release of drugs through the dissociation of polymer matrix at low pH prevailing in the endo-lysosomal complex, its induce ultrastructural changes in the cells due to cytotoxic effects associated with drugs. The red arrows in the micrographs indicate the distribution of CPMSNs in various organelles and green arrows specify damaged segments of the nucleus, ER, and mitochondria of both breast cancer apoptotic cells. At 4 h, the spherical shaped CPMSNs were seen to be slightly distributed in the cytoplasm, nucleus, ER, and mitochondria the without any morphological change. Images of 12 h treatment of MDA-MB-231 cells show accumulation of large amounts of CPMSNs with slight changes in morphology of the nucleus, ER, and mitochondria; however, images of 24 h treatment show pathological features such as membrane damage in the ER and mitochondria and condensation of nuclei due to release of high quantity of drugs from CPMSNs. Though both the treated cancer cells showed the events of accumulation of CPMSNs and damage of organelles, the events were less in MCF-7 cells than MDA-MB-231 cells suggesting the facilitation of entry of CPMSNs through integrin mediated endocytosis. Mesoporous silica nanoparticles facilitate its cellular uptake and aggregation in different subcellular organelles of cancer cells^28^. Studies on nanotherapeutic and theranostic materials having the ability to target and enter cancer cells through receptor mediated endocytosis and distribute drugs into cytoplasm after getting processed in endosomes and lysosomes^56^,^57^,^58^. Additionally, these kind of cellular internalization behaviour and viability was found to vary with different cell types due to its receptor expressions. These results are in agreement these developed nanocarriers may be able to serve as guidelines for tailoring polymeric nanovehicles for specific desired applications in biological and pharmaceutical fields, including the design of nanometer to submicron-sized delivery vehicles against breast cancer. In otherwise the controlled hierarchical organization of nanomaterials and their pH dependant biological performance opens up new horizons for exploring next-generation cRGD-based drug delivery systems with improved efficacy^59^,^60^,^61^.

### Western blot analysis

**Figure 9:**
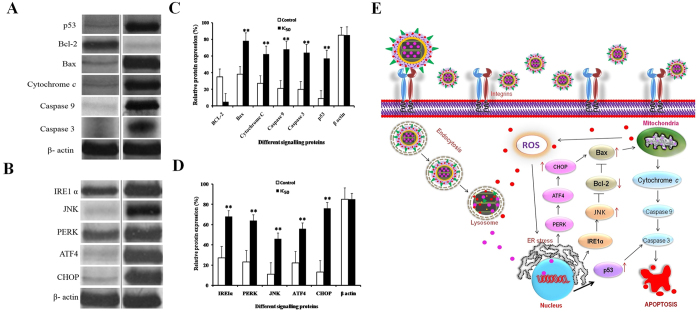
Western blot analysis of expression level of apoptotic and anti-apoptotic proteins by MDA-MB-231 cells treated with CPMSNs (**A–D**). ER and mitochondrial membrane proteins along with p53 were found to be overexpressed in CPMSNs treated cells when compared to untreated cells. However, the expression of mitochondrial membrane protein Bcl-2 was found to be down regulated in treated cells, indicating the induction of mitochondrial dependant apoptosis. β-actin served as internal standard. (**E**) The schematic representation of molecular mechanisms involved in the induction of apoptosis in MDA-MB-231 cells while CPMSN targets the integrin receptors via interaction with cRGD to facilitate internalization of CPMSNs by cells. The intracellular acidic pH triggers rapid dissociation of nanocarrier resulting in discharge of TPT and QT drugs. The released drugs induce ER stress, activate p53 and promote loss of Δψm leading to apoptosis.

The molecular mechanism involved in the apoptosis of CPMSNs treated MDA-MB-231 breast cancer cells (Fig. 9) was studied by Western blot analysis, wherein the expression profiles of ER stress proteins, apoptotic and anti-apoptotic proteins such as inositol-requiring 1 (IRE1α), c-Jun N-terminal kinase (JNK), PKR-like endoplasmic reticulum kinase (PERK), activating transcription factor 4 (ATF4), C/EBP homologous protein (CHOP), p53, Bcl-2, Bax, Cytochrome c, Caspase-9 and Caspase-3 were analyzed. It was observed that the cellular apoptotic genes such as p53, JNK, CHOP, Bax, Cytochrome c, caspase-9 and caspase-3 were upregulated in the treated cells, whereas the anti-apoptotic gene bcl-2 was down-regulated (Fig. 9A–D). The housekeeping gene β-actin served as an internal control and its expression remained unaltered.

Apoptosis is regulated by a dynamic interaction between pro-and antiapoptotic members of the Bcl-2 family proteins (Bcl-xl, Bcl-w, Mcl-1, A1)^62^,^63^. which are essential for controlling the mitochondrial outer membrane permeabilization (MOMP)^64^,^65^,^66^. Destabilization of the mitochondrial integrity by genotoxic as well as cytotoxic agents pave the way for activation of caspases to induce apoptosis. In our study, CPMSNs up-regulated the expression level of p53 gene, which in turn enhanced the expression of Bax. The insertion of Bax into the mitochondrial membrane, possibly leads to p53-mediated apoptosis. CPMSNs also elicited the expression of ER-stress-induced cell-death modulator such as Oligomerized IRE1 and PERK activated downstream signaling kinase JNK leads to the activation of transcription factors such as ATF4 and CHOP (also known as GADD153), resulting in dysfunction of mitochondria. Activation of pro-apoptotic Bax expression and decreased Bcl-2 expression affect the MOMP by inducing changes in mitochondrial permeability transition pore^65^,^67^. This results in the translocation of Bax from cytosol to mitochondria leading to the release of cytochrome c, a pro-apoptotic molecule into the cytoplasm *via* formation of pores. The released cytochrome c activates the caspase-3 and 9, key factor both in the initiation and execution of apoptosis and also accountable for the cellular DNA fragmentation during apoptosis^68^,^69^,^70^,^71^. An up-regulation in the expression of cytochrome c, caspase-9 and caspase-3 suggests their role in the apoptosis in CPMSNs treated cells. Our results in expression of apoptotic proteins were in correlation with the previous studies^72^. Figure 9E shows the schematic representation of molecular mechanisms involved in the induction of apoptosis of breast cancer cells while the integrin receptors are interact with cRGD peptides of the constructed CPMSN nanocarriers.

### *In vivo* anti-tumor activity

**Figure 10:**
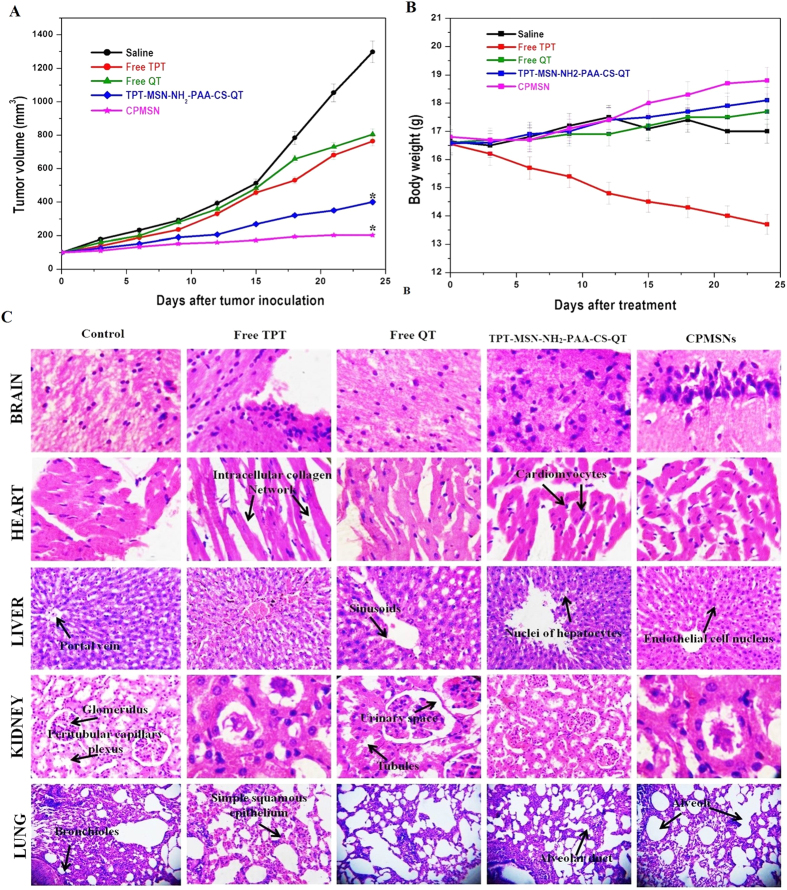
*In vivo* combination therapy. (**A**) Change in the tumor volume and (**B**) weight of mice in five different treatment groups. Error bars are based on standard error of the mean. *p ≤ 0.05 was considered statistically significant; (**C**) Histopathological studies on anti-tumor effects of free TPT, free QT, TPT-MSN-NH_2_-PAA-CS-QT, and CPMSNs in mice injected with MDA-MB-231 cells. The figures show that CPMSNs are nontoxic as the nanoformulations do not cause any damage to the vital organs; however, the formulations show a remarkable reduction in tumor volume.

The mice experiments were carried out in Sankaralingam Bhuwaneswari College of Pharmacy “accordance” with the relevant guidelines of CPCSEA with the regulatory approval (622/PO/C/02/CPCSEA, dated 27.01.2014). In order to evaluate the tumor targeting capability and drug distribution of nanomaterials *in vivo*, the normal saline, free TPT, free QT, TPT-MSN-NH_2_-PAA-CS-QT and CPMSNs were injected at a dose of 5 mg/kg body weight through the tail vein of the mice bearing breast carcinoma MDA-MB-231 cells and the antitumor activity of nanoparticles was assessed. The tumor volume was recorded for a period of 24 days after the start of treatment, and mice injected with saline were treated as the control group. Figure 10A shows the tumor growth curves after 24^th^ day of treatment. The tumor volume of mice receiving normal saline rapidly increased at the end of the 24^th^ day and was observed to be 1297.33 ± 0.11 mm^3^. There was no significant difference of the tumor inhibition rate among the groups treated with normal saline. Mice treated with free TPT and free QT showed modest tumor growth inhibition compared to the saline group with tumor volume of 765.05 mm^3^ and 805 mm^3^ respectively. The tumor volume of mice treated with TPT-MSN-NH2-PAA-CS-QT was 400 mm^3^. However, tumor inhibition was significant in the groups treated with CPMSNs (202 mm^3^). The enhanced antitumor activity of CPMSNs was attributed to improved internalization and accumulation of drugs by cells through targeted nanocarriers and pH controlled release of drugs. The relatively weak antitumor effect of TPT-MSN-NH2-PAA-CS-QT suggested the importance of targeting properties of CPMSNs. Though the volume of tumor is reduced after CPMSN treatment, it is critical to evaluate the loss of animal body weight. The fluctuation in animal body weight is recognized as an useful indicator to assess *in vivo* toxicity of drug delivery systems. As can be seen in Fig. 10B, mice administrated with saline showed a steadily increasing body weight. Similarly, mice administered with free QT, TPT-MSN-NH_2_-PAA-CS-QT and CPMSN also exhibited no decline in body weight, indicating the nontoxic nature of the QT and nanomaterials. In contrast, the body weights of free TPT treated group of animals was sharply decreased compared to control and other formulations suggesting that TPT could induce adverse effects of the given dose. Moreover, histological analysis of H&E stained slices of organs, including brain, heart, kidney, liver and lung recovered from mice treated with normal saline, free QT, TPT-MSN-NH_2_-PAA-CS-QT and CPMSNs revealed no significant damage or pathological features (Fig. 10C). However, the mice group treated with free drug TPT showed more acute inflammatory cell infiltration with obvious organ damage of necrosis in heart and kidney tissues than in control mice. These findings indicated that the drug TPT was delivered not only in tumor cells but also to other normal cells and caused adverse effects. Though several chemical modifications were carried out in the preparation of multifunctional MSN materials so as to provide the desired properties such as nontoxicity, biodegradability, biocompatibility and targeted drug delivery, shelf life of the materials in circulation, degradation and excretion are the concerns for safe use of the nanocarriers. There are studies suggesting the biocompatible nature of MSNs *in vivo* within desirable dose and they can be easily degraded and expelled from the body via urine and feces^73^,^74^,^75^. There remain many challenges that need to be overcome since the *in vivo* therapeutic use of CPMSNs require more validation and performance studies *in vivo* to satisfy the complicated physiological environment so as to avoid the undesired effects of MSNs. It is expected that the drugs TPT and QT would remain on the CPMSNs through a pH dependant mechanism for a considerable duration in the blood circulation (pH 7.4), which greatly reduces the exposure of drugs to normal tissues and thus decreased the toxicity and adverse effects of drugs while effectively enhancing its anticancer activity.

## Conclusion

In this study, an integrin receptor targeted nanocarriers (CPMSNs) have been synthesized for combination therapy against breast cancer. A pH-responsive MSN-based nanocarrier system for dual drug delivery was developed by using MSNs as core structure and biocompatible PAA-CS polymer shell. The assembled polymer shell not only provides the pH sensitivity and improved biocompatibility, but also facilitates further functionalization of MSNs with multifunctional ligand molecules (cRGD). The functionalized nanocarrier CPMSN exhibited remarkable pH-responsiveness, capability to target cancer cells, intracellular accumulation and sustained drug release and induction of apoptosis in both Triple Negative Breast Cancer (TNBC) and Multidrug resistant breast cancer cells as substantiated by *in vitro* and *in vivo* studies. These findings desirable the chemotherapeutic features of CPMSNs as a promising strategy for treating breast cancer with high clinical outcome.

## Materials and Methods

### Materials

Cetyltrimethylammonium bromide (CTAB), Tetraethyl orthosilicate (TEOS), 3-Aminopropyl triethoxysilane (APS), Poly acrylic acid (PAA), and Chitosan (CS) were purchased from Hi Media, India. Topotecan hydrochloride hydrate (TPT), Quercetin (QT), 3-(4,5-dimethylthiazol-2-yl)-2,5-diphenyltetrazolium bromide (MTT), 1-Ethyl-3-(3-dimethylaminopropyl) carbodiimide (EDC), N-Hydroxysuccinimide (NHS), and 2′,7′-Dichlorofluorescein diacetate (DCFH-DA) were purchased from Sigma-Aldrich, India.

### Preparation of Mesoporous silica nanoparticles

In a typical reaction^16^, 0.1 g CTAB, 200 mL H_2_O, and 1 mL NaOH (2 mM) were mixed with stirring and heated at 80 °C for 2 h. Later, 0.5 mL of TEOS was added drop wise into reaction mixer and the reaction mixture was kept at 80 °C for 6 h. The resultant white precipitate was collected by centrifugation at 10,000 rpm for 10 min. To remove the surfactant from the nanoparticles, 2 mL HCl (0.1%) and 20 mL of MeOH was added and then the solution was heated at 80 °C for 12 h. The surfactant was eliminated by centrifugation, rinsed 3 times with ethanol, and finally dried overnight in a vacuum at 60 °C for 12 h. Then, MSN (0.1 g) was dissolved in 20 mL of anhydrous ethanol, heated to 80 °C for 5 min. APS (1 mL) was added into the solution to functionalize the MSN with amino groups. The reaction mixture was refluxed for 24 h, and it was cooled down to room temperature followed by centrifugation, washed 3 times with ethanol and H_2_O, and dried overnight in a vacuum at 60 °C for 12 h to give MSN-NH_2_ as a white powder.

### Drug loading efficiency of TPT

To load anticancer drug TPT onto MSN-NH_2_, 6 mL of MSN-NH_2_ dispersion (1 mg/mL^−1^) was added with a 2 mL aqueous solution of TPT (2 mg/mL^−1^) and the mixture was stirred for 12 h at room temperature. The nanoparticle (TPT-MSN-NH_2_) were gathered through centrifugation and carefully rinsed 3 times with ethanol to remove the surface adsorption of TPT. The loading efficiency of TPT was determined by measuring the adsorption of the supernatant using UV/Vis spectrophotometer at 381 nm^2^.

### Synthesis of PAA-CS coated TPT-MSN-NH_2_

The TPT-MSN-NH_2_ was dispersed (20 mg) in a 15 mL mixture of DMF and H_2_O containing 0.15 mg of PAA (Mw = 18 000) and 0.35 g of CS and the mixture was stirred at 50 °C for 6 h. The resultant product was centrifuged, washed several times with deionized water to remove non-conjugated PAA-CS and dried overnight in a vacuum at 45 °C.

### Loading of QT and grafting of cRGD on TPT-MSN-NH_2_-PAA-CS

QT was suspended (25 mg) in 50 mL of aqueous solution containing 0.2 g of TPT loaded MSN-NH_2_-PAA-CS. The product was stirred at 45 °C for 24 h followed by washing with water several times to remove unloaded QT. In this preparation, 300 mg of EDC and 250 mg of NHS was added and further stirred at room temperature for 24 h. Subsequently, the solution was added to 0.01 mg of cRGD peptide and incubated for 2 h at room temperature to obtain CPMSNs. The adsorption measurements of the original solution and supernatant were recorded to determine the amount of QT that was loaded in CPMSN, and the loading efficiency was calculated at 375 nm using UV-Vis spectrophotometer.

### Characterization of synthesized CPMSN

The morphology and mesoporous aperture of the nanoparticles were analyzed by scanning electron microscope (SEM), (SEM, FEI-QUANTA 200) and transmission electron microscope (TEM) (TECNAI G2, with an acceleration voltage of 200 kV). The structure of nanoparticles was investigated by Fourier transform infrared spectroscopy (FTIR; BRUKER VECTOR22,) in the scanning range of 4000–400 cm^−1^ using KBr pellet technique. The size distribution of the nanoparticles was measured by dynamic light scattering (DLS) using a Master sizer 3000. N2 adsorption–desorption isotherms were measured with an automatic surface area and porosity analyzer (3H-2000PS2, Beishide) at 77 K. Thermogravimetric analysis (TGA) was performed using a Perkin Elmer PYRIS 1 DSC at a heating rate of 10 °C min^−1^ in a nitrogen flow with temperature ranging from 0 to 700 °C. The Brunauer–Emmett–Teller (BET) method was utilized to calculate the specific surface areas using adsorption data in a relatively pressure range from 0.05 to 0.95. The pore volumes and pore size distributions were derived from the desorption branches of the isotherms using the Barrett–Joyner–Halanda (BJH) method. The zeta potential of the nanoparticles was measured using a zeta potential analyzer (Nanotrac wave, Microtrac) at 25 °C with DI water as the solvent. The pH-responsive releases of the drugs were detected by measuring their absorbance at 381 nm and 375 nm is using UV-vis spectrophotometer (LS50B, PerkinElmer).

### *In vitro* release of TPT and QT

The CPMSNs (2 mg) were put into the dialysis bag (molecular weight cut off 7000) and dipped into 50 mL of PBS solution with different pH levels (7.4, 6.0, and 5.0), and incubated at 35 °C. At given time intervals (10 min), 200 μL of the solution was withdrawn and the original volume was restored with supplement of the fresh PBS solution. The amounts of the liberated drugs were measured at 381 nm and 375 nm for TPT and QT respectively by UV-Vis spectrophotometer.


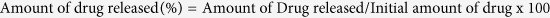


### Cell culture

Human breast epithelial cell line (HBL-100) and Human breast cancer cell lines MDA-MB-231 and MCF-7 cells were purchased from National Centre for Cell Sciences (NCCS), Pune, India. These cell lines were maintained in DMEM media supplemented with 10% FBS, 1% L-glutamine, 1% penicillin and 1% streptomycin at 37 °C in 5% CO_2_ humidified incubator. Media were changed every two days, and the cells were passaged by trypsinization.

### Cell viability assay

The HBL-100 cells (Control) and MDA-MB-231 and MCF-7 (Experimental) cells were plated in 12 well plates at a density of 2 × 10^5^ cells/well and grown for 24 h. The Control cells were treated with various concentration (1, 2, 4, 8, 16, 32 μg/mL) of free TPT, free QT, free TPT+QT, TPT-MSN-NH_2_-PAA-CS-QT and CPMSNs for 24 h, whereas experimental cells were treated with different concentration (0.125, 0.250, 0.5, 1.0, 1.5 and 2.0 μg/mL) of free TPT, free QT, free TPT+QT, TPT-MSN-NH_2_-PAA-CS-QT and CPMSNs for 24 h. The cell viability was calculated as a percentage of viable cells after treated with drug or drug loaded nanoparticles compared with the untreated cells.

### Analysis of cellular uptake efficacy of CPMSN nanocarriers by confocal microscopy

The MDA-MB-231 and MCF-7 breast cancer cells were grown in 6-well plates (1 × 10^4^ cells/well) for 24 h and were treated with CPMSNs at IC_50_ concentration for 4 h, 12 h and 24 h. The cells (1 × 10^5^ cells/coverslip) were washed thrice with ice cold PBS, fixed in methanol: acetic acid (3:1 v/v) and stained with DAPI at 37 °C for 30 min. The cells were again washed with PBS and observed using confocal fluorescence microscope (Nikon, Japan).

### DAPI and Rhodamine 123 staining

In another experiment, both the experimental cells were cultured in 6-well plate (2 × 10^6^ cells/well), treated with free TPT, free QT, free TPT+QT, TPT-MSN-NH_2_-PAA-CS-QT and CPMSNs for 24 h and stained with DAPI as described previously^3^. Besides, these cells were also treated with 5 μg/mL of Rhodamine 123, washed with PBS and examined using a fluorescence microscope (FLoid™ Cell Imaging Station, THERMO FISHER).

### Measurement of ROS Generation

The levels of intracellular peroxide and superoxide free radicals were measured by adding 5 μg/mL of 2′, 7′-Dichlorofluorescein diacetate (DCFH-DA) to 6-well plate containing 5 × 10^5^ cells/well, and treated with final concentration (2 μg/mL) of free TPT, free QT, TPT+QT, TPT-MSN-NH_2_-PAA-CS-QT, and CPMSN for 24 h in a 37 °C incubator. The fluorescence intensity of DCF is proportional to the amount of ROS produced by the cell. ROS generation was assessed using a fluorescence microscope (Nikon Eclipse, Inc., Japan) at excitation and emission wavelengths of 488 and 530 nm, respectively, and the mean fluorescence intensity of DCF was evaluated.

### TEM studies on cellular internalization of CPMSN

The MDA-MB 231 cells were treated with CPMSNs as stated earlier followed by washing with phosphate buffer to remove the unbound CPMSNs. The cells were then fixed in 2% glutaraldehyde for 30 min at 4 °C. Fixed cells were collected, washed thrice with phosphate buffer and dehydrated in increasing concentrations of acetone (40, 60, 80 and 100%) followed by treatment with spur’s low viscosity resin (Sigma Aldrich, USA) in gradients. Acetone and spur’s low viscosity resin were used in 3:1, 1:1 and 1:3 ratios. Finally, 100% spur resin was added and the beam capsule was incubated for 80 h at 70 °C. The cell sections of 60 nm thickness were obtained using microtome, stained with 0.5% of uranyl acetate and examined by TEM to observe the distribution of CPMSNs.

### Real Time PCR (RT-PCR) analysis for gene expression

RT-PCR was used to analysis the integrin expression profile in breast cancer cells. For this, total RNA was isolated from both cancer cells using TRIzol Reagent (Sigma Aldrich, Bangalore). It was subjected to the RT-PCR using the sequences of the sense and anti sense primers were as follow: integrin forward: 5′-AGGATAGTTCTGTTTCCTGC-3′: and reverse: 5′-CCTGGAAGATGGTGATGGGAT-3′:GAPDH; 5′-AACGGATTTGGTCGTATTGGG-3′, and reverse 5′-CCTGGAAGATGGTGATGGGAT-3′; relative gene expression level was normalized to GAPDH as a control, All experimental steps were followed through according to the previous report^76^.

### Western blot analysis

The cells were treated for 24 h with CPMSN at 2.0 μL final concentration for the induction of apoptosis, harvested, and rinsed twice with ice-cold PBS. 1.5 mL microfuge tube containing these cell extracts were centrifuged at 12000 rpm for 10 min at 4 °C, and the supernatants were stored at −80 °C until further use. The cells were washed in PBS and lysed in 100 μL of buffer containing 50 mM Tris-HCL (pH 8.0), 150 mM NaCl, 1% Triton X-100, 1 mM phenylmethane-sulfonyl fluoride and 10 μg/mL leupeptin. Proteins (30 μg/lane) were separated using 10% SDS-PAGE and transferred to PVDF membranes. The membrane was blocked in tris-buffered saline and tween 20 (TBST) solution containing 5% (w/v) non fat milk for 2 h, followed by overnight incubation at 4 °C with primary antibodies such as p53, IRE1α, JNK, PERK, ATF 4, CHOP, Bax, Bcl-2, Cytochrome c, Caspase 9, Caspase 3 and β-actin. The blots were washed with washing buffer and incubated with secondary antibodies conjugated with horseradish peroxidase for 1 h at room temperature.

### *In vivo* anticancer efficacy

All the animal experimental procedures were approved by the Institutional Animal Ethical Committee (IAEC) meeting at Sankaralingam Bhuwaneswari College of Pharmacy (622/PO/C/02/CPCSEA, dated 27.01.2014) and performed in accordance with standard animal handling and research practice. Female athymic nude mice of 5–6 week old weighing around 18–21 g were injected 5 × 10^6^ MDA-MB-231 cells subcutaneously. After 8 days, the mice were randomly divided into five groups (four mice per group) and were administrated with a weekly IV injection of saline, free TPT (5 mg/kg), free QT (5 mg/kg), TPT-MSN-NH_2_-PAA-CS-QT (2.5 mg/kg of TPT and 2.5 mg/kg of QT) and CPMSN (2.5 mg/kg of TPT and 2.5 mg/kg of QT). Thereafter, the tumor volume and body weight were monitored for 24 days. Tumor volume was calculated using the following formula: TV = (L-W^2^)/2, with W being smaller than L. After these treatments, mice were sacrificed using a CO_2_ inhalation method and the major organs such as brain, heart, liver, kidney and lung were excised and immediately fixed in 10% formalin in PBS solution. The organs were embedded in paraffin, cut into 4 μm sections using microtome, and then placed onto the glass slides. These sections were stained with hematoxylin and eosin (H&E) and histological images were obtained using a Nikon Eclipse 90i light microscope.

### Statistical Analysis

The data are shown as the mean ± standard deviation (SD) of the experiments performed in triplicates. Statistical comparisons were carried out using Student’s t test. p < 0.05 was considered to be significant. The intensity of the bands on the membrane was analyzed using the Bio-Rad image analysis system with Image-Pro software (Bio-Rad laboratories Inc., Hercules, CA, USA).

## Additional Information

**How to cite this article**: Murugan, C. *et al.* Combinatorial nanocarrier based drug delivery approach for amalgamation of anti-tumor agents in bresat cancer cells: an improved nanomedicine strategies. *Sci. Rep.* **6**, 34053; doi: 10.1038/srep34053 (2016).

## Supplementary Material

Supplementary Information

## Figures and Tables

**Table 1:** Zeta potential analysis values of synthesized nanomaterials at pH 6.8.

Samples	Zeta Potential (in pH 6.8)^a^/mV
MSNs	−20.40
MSN-NH_2_	+16.40
CPMSNs	+42.80

^a^Nano-formulations were dissolved in double distilled water.
