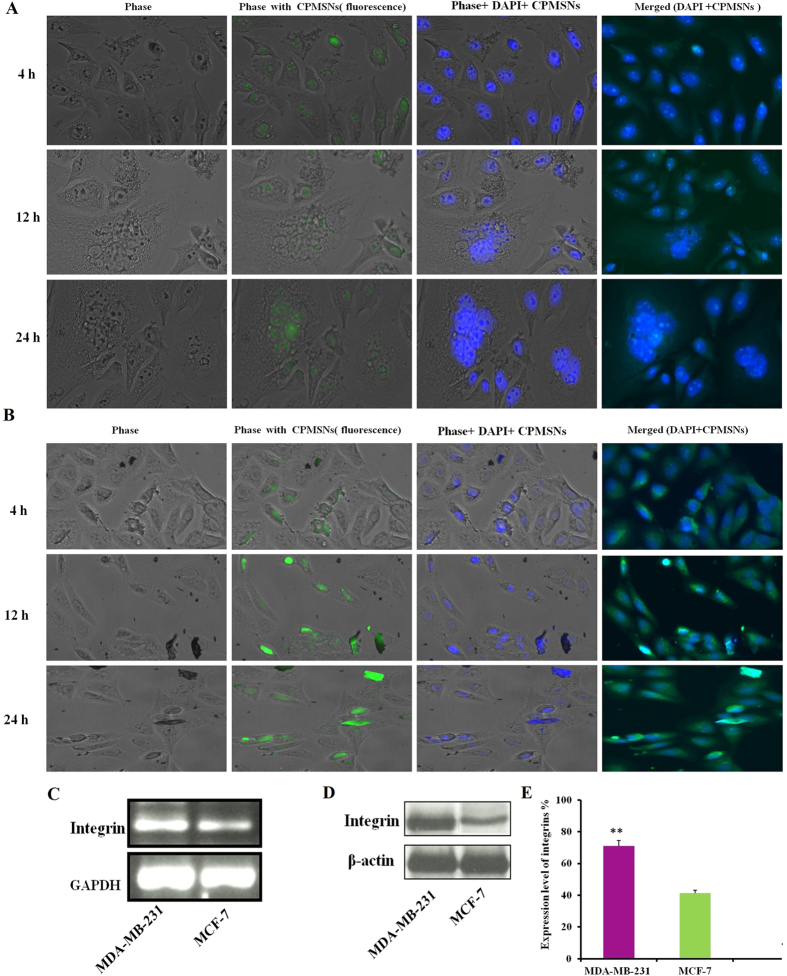# Corrigendum: Combinatorial nanocarrier based drug delivery approach for amalgamation of anti-tumor agents in breast cancer cells: an improved nanomedicine strategy

**DOI:** 10.1038/srep38146

**Published:** 2016-11-29

**Authors:** Chandran Murugan, Kathirvel Rayappan, Ramar Thangam, Ramasamy Bhanumathi, Krishnamurthy Shanthi, Raju Vivek, Ramasamy Thirumurugan, Atanu Bhattacharyya, Srinivasan Sivasubramanian, Palani Gunasekaran, Soundarapandian Kannan

Scientific Reports 6: Article number: 3405310.1038/srep34053; published online: 10 11 2016; updated: 11 29 2016

The original version of this Article contained errors. The Title,

“Combinatorial nanocarrier based drug delivery approach for amalgamation of anti-tumor agents in bresat cancer cells: an improved nanomedicine strategies”.

now reads:

“Combinatorial nanocarrier based drug delivery approach for amalgamation of anti-tumor agents in breast cancer cells: an improved nanomedicine strategy”.

Affiliation 1 was incorrectly listed as ‘Proteomics and Molecular Cell Physiology Laboratory, Department of Zoology, Periyar University, Salem-636011, TamilNadu, INDIA’. The correct affiliation is listed below:

‘Proteomics and Molecular Cell Physiology Laboratory, Department of Zoology, Periyar University, Salem-636011, Tamil Nadu, INDIA’.

In the Abstract,

“Subsequently, grafting of arginine-glycine-aspartic acid (cRGD) peptide on the surface of nanocarrier (CPMSN) thwarted the uptake by normal cells, but facilitated their uptake in cancer cells through integrin receptor mediated endocytosis and the dissociation of nanocarriers due to the ability to degrade of CS and PAA in acidic pH, which enhance the intracellular release of drugs”.

now reads:

“Subsequently, grafting of arginine-glycine-aspartic acid (cRGD) peptide on the surface of nanocarrier (CPMSN) thwarted the uptake by normal cells, but facilitated their uptake in cancer cells through integrin receptor mediated endocytosis and the dissociation of nanocarriers due to the ability to degrade CS and PAA in acidic pH, which enhance the intracellular release of drugs”.

In the Results and Discussion section under subheading ‘Synthesis and Characterization of CPMSN.’,

“The FT-IR spectra of MSN-CTAB, MSN, MSN-NH_2_, TPT-MSN-NH_2,_ TPT-MSN-NH_2_-PAA-CS, TPT-MSN-NH2-PAA-CS-QT and CPMSN are shown in Figure S1”.

now reads:

“The FT-IR spectra of MSN-CTAB, MSN, MSN-NH_2_, TPT-MSN-NH_2,_ TPT-MSN-NH_2_-PAA-CS, TPT-MSN-NH-PAA-CS-QT and CPMSN are shown in Figure S1”.

In the same section under subheading ‘Drug Loading Profile of CPMSNs.’,

“The drug loading content of CPMSN (mass of drug in the CPMSNs/mass of CPMSNs loaded with drug ×100%) and encapsulation efficiency (mass of drug achieved in the NCs/mass of the feeding drug × 100) 0.010/0.014 × 100 = 71.4% of TPT and QT 0.012/0.018 × 100 = 66.6%) of TPT and QT in CPMSN were calculated as 1.8, 1.2 wt%, respectively”.

now reads:

“The drug loading content of CPMSN (mass of drug in the CPMSNs/mass of CPMSNs loaded with drug ×100%) and encapsulation efficiency (mass of drug achieved in the NCs/mass of the feeding drug × 100) 0.010/0.014 × 100 = 71.4% of TPT and QT 0.012/0.018 × 100 = 66.6%) of TPT and QT in CPMSN were calculated as 1.8 wt%, 1.2 wt%, respectively”.

In the same section under subheading ‘*In vitro* Drug Release.’,

“Subsequently, it enhances the release of QT from polymer and TPT release from the pores of MNPs as well”.

now reads:

“Subsequently, it enhances the release of QT from polymer and TPT release from the pores of MSNs as well”.

In the same section under subheading ‘Analysis of Δψm loss and nuclear morphology by fluorescence microscopy’,

“It was observed that the growth of cells was inhibited when they were treated with free TPT, free QT, free TPT+ QT, TPT-MSN-NH2-PAA-CS-QT, and CPMSNs at their final concentrations (2 μg/mL), and the CPMSNs possess higher antiproliferative efficacy against cancer cells than free drugs”.

now reads:

“It was observed that the growth of cells was inhibited when they were treated with free TPT, free QT, free TPT+ QT, TPT-MSN-NH-PAA-CS-QT, and CPMSNs at their final concentrations (2 μg/mL), and the CPMSNs possess higher antiproliferative efficacy against cancer cells than free drugs”.

and,

“Fluorescence images of cancer cells show that there was a reduction in the mean fluorescence intensity for cells treated with free TPT, free QT, free TPT+QT, TPT-MSN-NH2-PAA-CS-QT, and CPMSNs confirming the loss of Δψm owing to mitochondrial membrane depolarization, which was considered to be an initial and irreversible step of apoptosis”.

now reads:

“Fluorescence images of cancer cells show that there was a reduction in the mean fluorescence intensity for cells treated with free TPT, free QT, free TPT+QT, TPT-MSN-NH-PAA-CS-QT, and CPMSNs confirming the loss of Δψm owing to mitochondrial membrane depolarization, which was considered to be an initial and irreversible step of apoptosis”.

In the same section under subheading ‘ROS generation in CPMSNs treated breast cancer cells.’,

“ROS generation were examined (Fig. 7A,B) in both cancer cells after treating them with free TPT, free QT, free TPT+QT, TPT-MSN-NH2-PAA-CS-QT and CPMSNs (2.0 μg/mL). ROS scavenger DCFH-DA stained treated MDA-MB-231 and MCF-7 cells showed increased fluorescence intensity when compared to untreated cells, suggesting that the chemotherapeutic drugs distributed by CPMSNs were able to ROS generation as well as DNA damage leading to induce apoptosis^53^. However, the TPT+QT drug combination did not show a statistically significant impact on ROS generation and this could be due to the antioxidant properties of QT. Though there was an increase in fluorescence intensity of cells treated with TPT-MSN-NH2-PAA-CS-QT and CPMSNs, cells treated with CPMSNs exhibited higher intensity of fluorescence than TPT-MSN-NH2-PAA-CS-QT”.

now reads:

‘ROS generation were examined (Fig. 7A,B) in both cancer cells after treating them with free TPT, free QT, free TPT+QT, TPT-MSN-NH-PAA-CS-QT and CPMSNs (2.0 μg/mL). ROS scavenger DCFH-DA stained treated MDA-MB-231 and MCF-7 cells showed increased fluorescence intensity when compared to untreated cells, suggesting that the chemotherapeutic drugs distributed by CPMSNs were able to ROS generation as well as DNA damage leading to induce apoptosis^53^. However, the TPT+QT drug combination did not show a statistically significant impact on ROS generation and this could be due to the antioxidant properties of QT. Though there was an increase in fluorescence intensity of cells treated with TPT-MSN-NH-PAA-CS-QT and CPMSNs, cells treated with CPMSNs exhibited higher intensity of fluorescence than TPT-MSN-NH-PAA-CS-QT’.

In the legend of Figure 2G,

“nitrogen adsorption desorption isotherms authenticate the porous nature of synthesized MSNs, MSN-NH_2_, and CPMSNs”.

now reads:

“nitrogen adsorption/desorption isotherms authenticate the porous nature of synthesized MSNs, MSN-NH_2_, and CPMSNs”.

The legend of Figure 3E was incorrectly labelled as “b”.

In Figure 5B, the y-axis was incorrectly labeled. The correct Figure 5B appears below as Figure 1.

The ‘How to cite this article:’ section,

“Murugan, C. *et al*. Combinatorial nanocarrier based drug delivery approach for amalgamation of anti-tumor agents in bresat cancer cells: an improved nanomedicine strategies. *Sci. Rep.* **6**, 34053, doi: 10.1038/srep34053 (2016)”.

now reads:

“Murugan, C. *et al*. Combinatorial nanocarrier based drug delivery approach for amalgamation of anti-tumor agents in breast cancer cells: an improved nanomedicine strategy. *Sci. Rep.* **6**, 34053, doi: 10.1038/srep34053 (2016)”.

These errors have now been corrected in the HTML and PDF versions of the Article.

## Figures and Tables

**Figure 1:**