# Supplementary material for: Combinatorial nanocarrier based drug delivery approach for amalgamation of anti-tumor agents in bresat cancer cells: an improved nanomedicine strategies
Source: Sci Rep. 2016 Oct 11;6:34053. doi: 10.1038/srep34053 (PMC5057072; doi:10.1038/srep34053)
Supplement: Supplementary Information [file srep34053-s1.pdf]

Supplementary Information

COMBINATORIAL NANOCARRIER BASED DRUG DELIVERY APPROACH FOR AMALGAMATION OF ANTI-TUMOR AGENTS IN BRESAT CANCER CELLS: AN IMPROVED NANOMEDICINE STRATEGIES

Chandran Murugan[1], Kathirvel Rayappan[1], Ramar Thangam[2], Ramasamy Bhanumathi[1], Raju Vivek[3], Krishnamurthy Shanthi[4], Srinivasan Sivasubramanian[2], Ramasamy Thirumurugan[5], Atanu Bhattacharyya[6], Palani Gunasekaran[2], and Soundarapandian Kannan[1*]

1. *Proteomics and Molecular Cell Physiology Laboratory, Department of Zoology, Periyar University,Salem-636011,TamilNadu,INDIA*
2. *King Institute of Preventive Medicine & Research, Guindy, Chennai 600 032, Tamil Nadu, INDIA.*
3. *Department of Biomedical Engineering, Shanghai Jiao Tong University, Shanghai-200 240, CHINA.*
4. *Department of Zoology, Bharathiar University, Coimbatore-641 046, Tamil Nadu, INDIA.*
5. *Department of Animal Science, Bharathidasan University, Tiruchirappalli – 620 024, Tamil Nadu, INDIA.*
6. *Nanotechnology Section, Department of Biomedical Engineering, Rajiv Gandhi Institute of Technology and Research Centre, Hebbal, Bangalore-560 032. Karnataka, INDIA.*

For Correspondence:

***E.mail:** sk_protein@periyaruniversity.ac.in

Supplementary Note 1: FT-IR spectroscopy of CPMSN

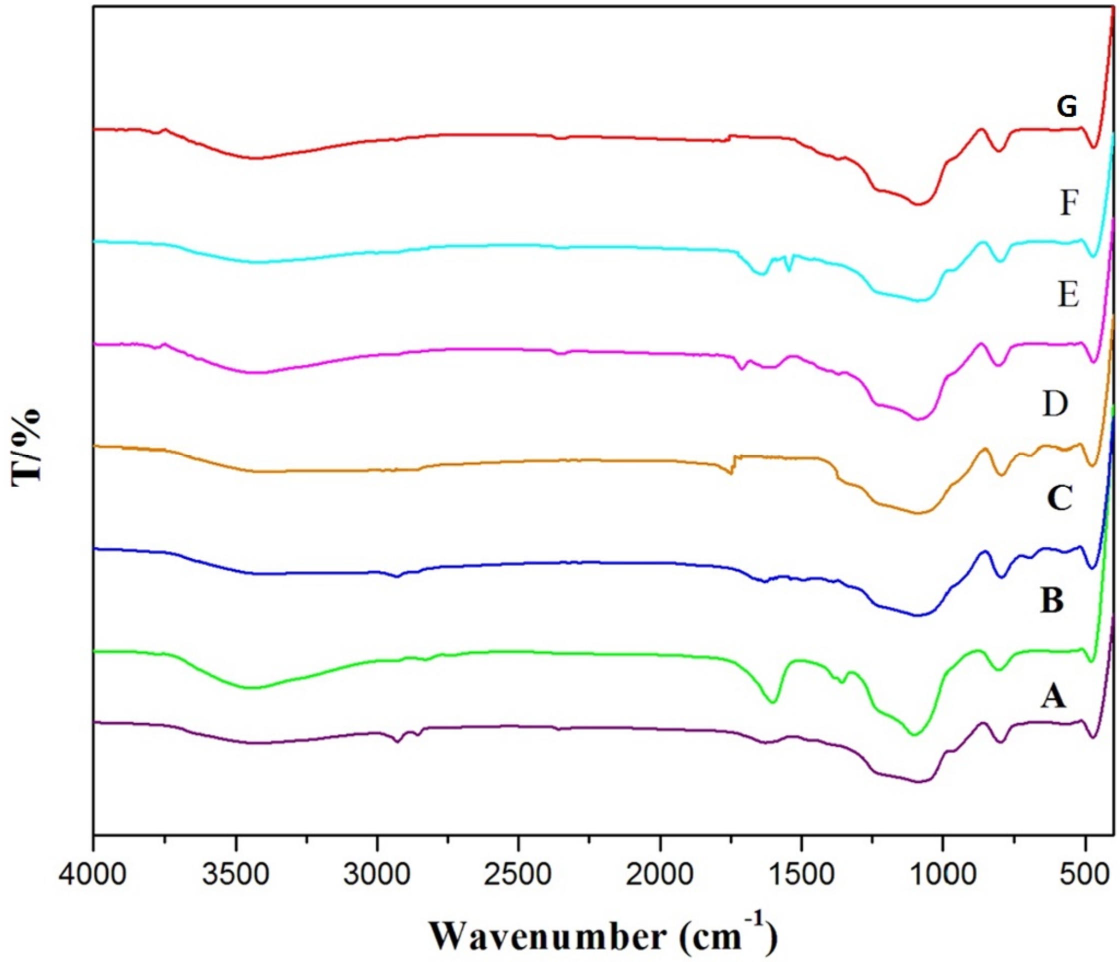

Figure S1. The FTIR spectrum of (A)CTAB (MSN-CTAB), (B) MSN, (C)MSN-NH_2, (D)TPT-MSN-NH_2, (E)TPT-MSN-NH_2-PAA-CS, (F)TPT-MSN-NH_2-PAA-CS-QT, and (G) CPMSN
